# Transforming Spinal Surgery: The Life and Innovations of Dr. Paul Harrington

**DOI:** 10.7759/cureus.71681

**Published:** 2024-10-17

**Authors:** Pankaj Sharma, Archit Gupta, SomiReddy Medapati

**Affiliations:** 1 Orthopaedics, Dr. D Y Patil Medical College, Hospital and Research Centre, Dr. D Y Patil Vidyapeeth (Deemed to be University), Pune, IND

**Keywords:** harrington rod, historical vignette, scoliosis research society, scoliosis surgery, spine deformity surgery, surgical innovator

## Abstract

This paper explores the life and contributions of Dr. Paul Harrington, a pioneering figure in spinal surgery, mainly known for his groundbreaking work in treating scoliosis. It delves into the development and impact of the Harrington rod, a revolutionary device that transformed the surgical management of spinal deformities. The article examines the historical context of scoliosis treatment before Harrington's innovations, the challenges he faced in developing his method, and the lasting influence of his work on modern orthopaedic surgery. Through an analysis of his life, the paper highlights Harrington's enduring legacy in spinal surgery and his role in advancing medical practices that continue to benefit patients worldwide.

## Introduction and background

Over the last century, spine surgery has seen remarkable advancements, particularly in treating spinal deformities such as scoliosis. These developments have significantly improved outcomes for children and adults suffering from conditions affecting the spine. Historically, before the availability of modern medications and vaccines for diseases like tuberculosis and poliomyelitis, patients often experienced debilitating spinal deformities, including kyphoscoliosis, as complications of these illnesses. The journey from these early struggles to the advanced surgical techniques we have today is marked by the contributions of several pioneering surgeons, most notably Dr. Paul Randall Harrington [1].

**Figure 1:**
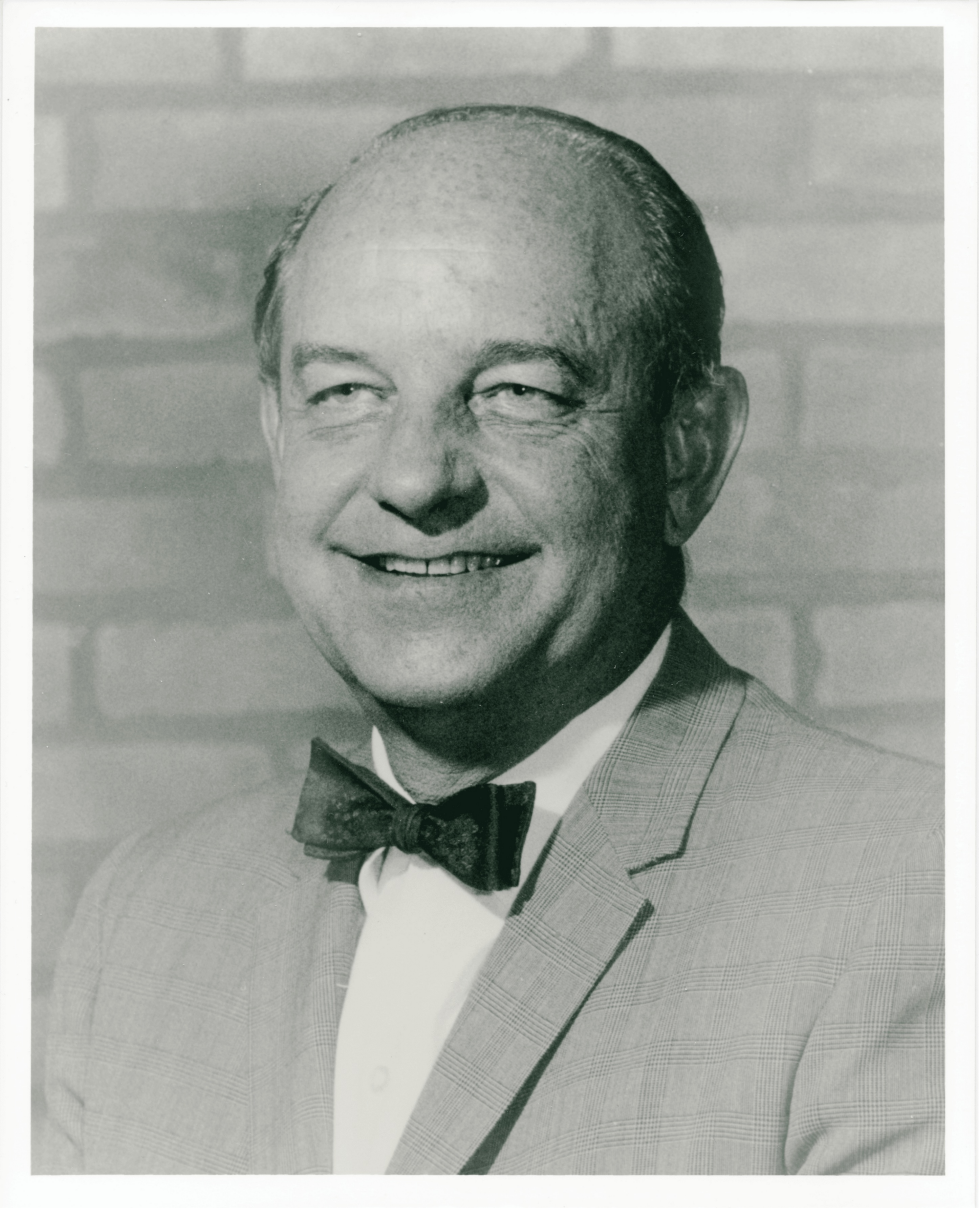
Dr. Paul Harrington, circa 1960 Credit: Paul R. Harrington Archives, University of Kansas Medical Centre, Kansas City, KS.

Today, Dr. Harrington is remembered as the 'Father of the Modern Treatment of Scoliosis' (Figure 1). His pioneering work has had a lasting impact on spinal surgery, and his innovations have inspired new generations of surgeons and researchers. Although no longer the standard treatment, the Harrington rod represents a significant milestone in the history of spinal surgery, and its development underscores the importance of innovation and dedication in advancing medical science. Dr. Harrington's contributions have improved countless patient lives and set the stage for ongoing advancements in the treatment of spinal deformities [2].

In the early 1900s, the treatment of spinal deformities began to take shape with efforts by surgeons like Dr. Russell A. Hibbs and Dr. Fred H. Albee. Dr. Hibbs performed the first spinal arthrodesis in 1911, a procedure designed to fuse the spine and correct scoliosis. Dr. Albee later adapted this technique to treat spinal deformities related to tuberculosis. These early procedures, while groundbreaking, faced significant challenges. Over time, they frequently led to a loss of correction, and patients often experienced pseudoarthrosis, a condition where the bone fusion fails to heal correctly [1].

The introduction of the Harrington rod, the first implantable spinal instrumentation system, by Dr. Paul Randall Harrington in 1947 marked a crucial turning point in the history of spine surgery. This innovation began a new era in treating scoliosis and other spinal deformities. Dr. Harrington's work revolutionized spinal surgery, particularly for patients with neuromuscular conditions such as poliomyelitis, where traditional treatments were often ineffective [2].

## Review

Early life and education

**Figure 2:**
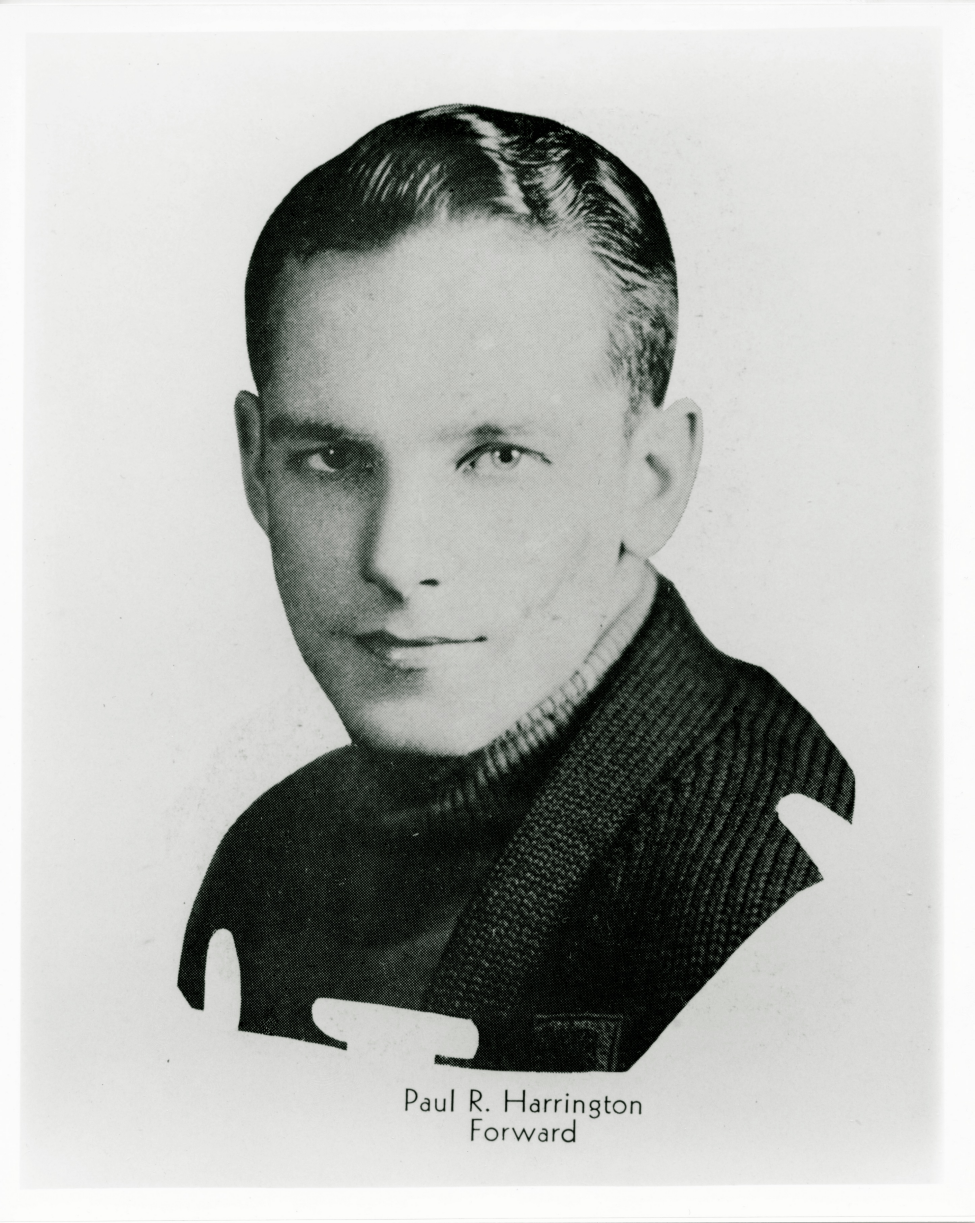
Dr. Paul Harrington basketball position photo, circa 1933-1934 Credit: Paul R. Harrington Archives, University of Kansas Medical Centre, Kansas City, KS.

**Figure 3:**
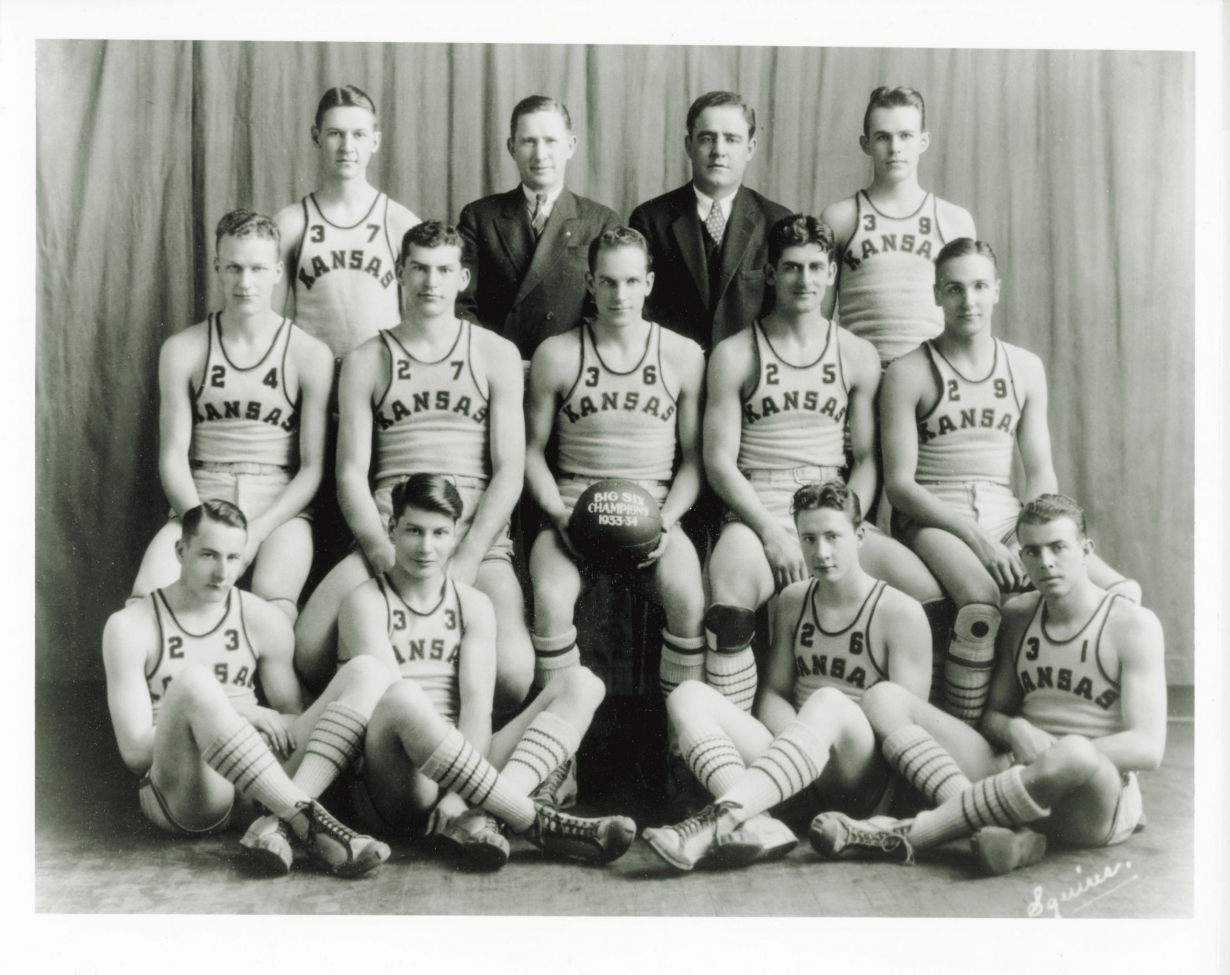
Dr. Paul Harrington (centre - holding the ball) basketball team group photo, circa 1933-1934 Credit: Paul R. Harrington Archives, University of Kansas Medical Centre, Kansas City, KS.

Dr. Paul Randall Harrington was born on September 27, 1911 in Kansas City, Kansas. He excelled both academically and athletically, particularly in basketball. He was given a scholarship at the University of Kansas because of his athletic ability, and was an important member of the basketball team (Figure 2). The team won the Big Six Conference championship for three consecutive years, and Harrington served as the team captain in his final year (Figure 3). His experience in athletics, which emphasized teamwork, perseverance, and leadership, would later significantly influence his approach to medicine and surgery [2].

After completing his undergraduate studies, Harrington pursued a medical degree, earning his MD from the University of Kansas School of Medicine in 1939. To finance his education, he played semi-professional basketball, demonstrating his commitment and resourcefulness. Following medical school, Dr. Harrington completed his surgical residency and internship at Charleston, South Carolina's Roper Hospital before starting his medical career. In 1942, he went back to Kansas City, Missouri, to do his residency in orthopaedic surgery at St. Luke's Hospital [3].

Early work

Dr. Harrington enlisted in the US Army Medical Corps during World War II, and from 1942 to 1945, he headed the orthopaedic surgery department at the 77th Evacuation Hospital. He gained experience during the war by treating soldiers in North Africa, Sicily, and Europe. This improved his surgical abilities and expanded his knowledge of orthopaedic problems and their treatment. It laid the foundation for his later innovations in spinal surgery [3].

After the war, Dr. Harrington moved to Houston, Texas, where his medical career took a decisive turn. He began working at Jefferson Davis Hospital, Houston's public hospital and developed a strong interest in treating patients with poliomyelitis, commonly known as polio. The period between 1944 and 1955 saw a significant polio epidemic in the United States, with over 10 cases per 100,000 people annually. A large percentage of those affected were children and many developed scoliosis as a result of the disease [3].

Dr. Harrington recognized the severe impact that neuromuscular scoliosis could have on patient health. Neuromuscular scoliosis is associated with conditions that affect the nervous system and muscles, such as poliomyelitis, spinal cord injuries, and cerebral palsy. This type of scoliosis progresses rapidly and can lead to significant functional impairments, resulting in decreased lung function and a lack of balance while sitting because of rib cage and spinal deformities [4].

Contribution to scoliosis treatment

Existing treatments for scoliosis at the time included physical therapy, bracing, and surgical methods developed by Drs. Hibbs, Albee, and Galloway. These were insufficient for treating youngsters whose polio had caused neuromuscular scoliosis. These patients needed a more effective and stable solution. Dr. Harrington's early efforts involved manually correcting scoliotic deformities and using internal fixation with facet screws. However, these methods did not provide long-term stability and the deformities often recurred [4].

Development of the Harrington rod system

**Figure 4:**
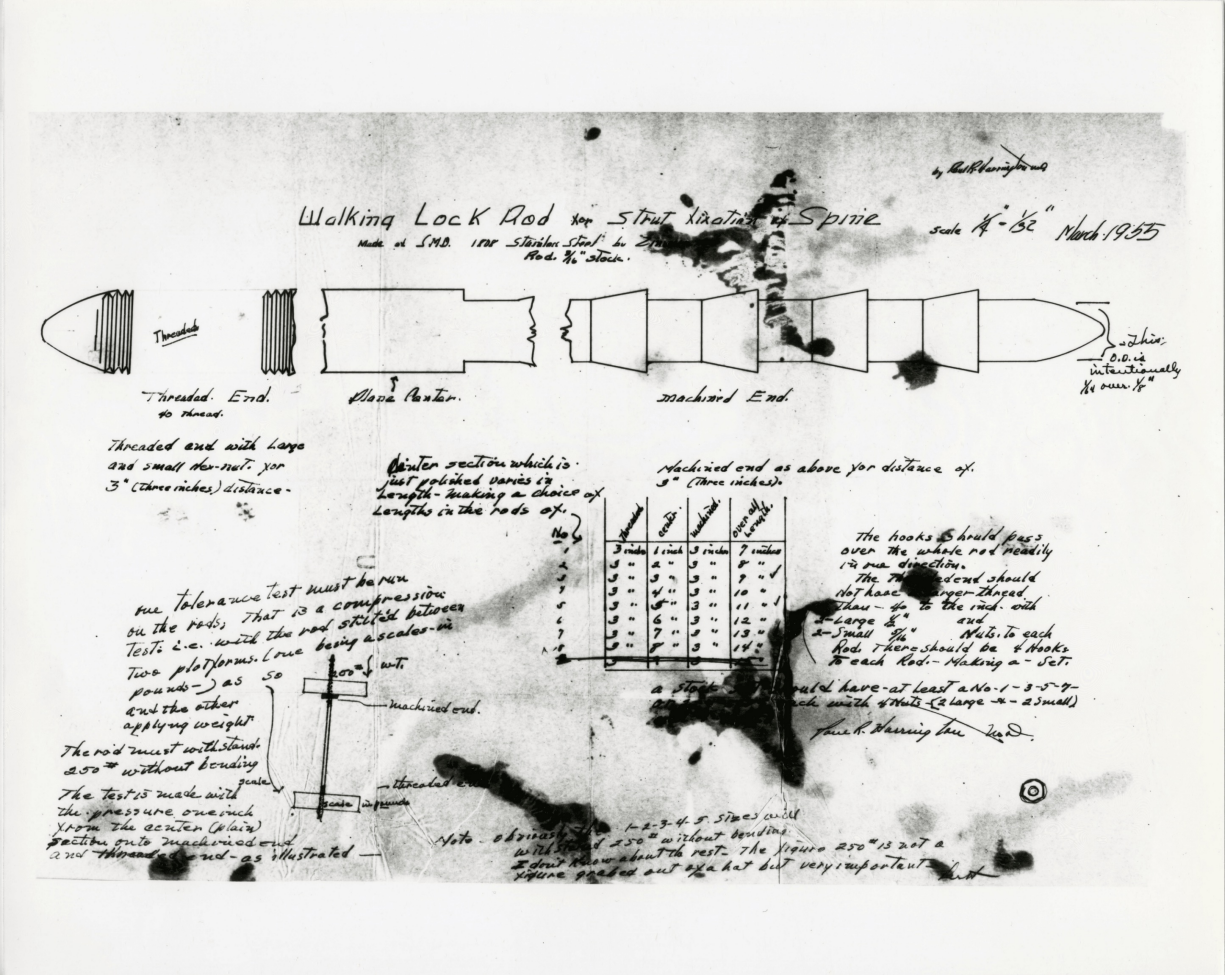
Early drawings of the walking lock rod system, circa 1955 Credit: Paul R. Harrington Archives, University of Kansas Medical Centre, Kansas City, KS.

To address this problem, Dr. Harrington teamed up with Thorkild Engen, an orthotist and engineer from Warm Springs, Georgia. They collaborated to create a new spinal instrumentation device using a hook and ratchet rod arrangement. This system, known as the Harrington rod, significantly advanced the treatment of spinal deformities (Figure 4). Dr. Harrington personally crafted the instruments before each surgery and adjusted them based on the outcomes observed in previous patients. This iterative approach allowed him to continually refine the design and improve the effectiveness of the treatment [4]. Although the introduction of the Salk vaccine reduced the incidence of post-poliomyelitis scoliosis, the Harrington rod system proved effective in treating various types of scoliosis. Dr. Harrington first presented his internal fixation method for the treatment of neuromuscular scoliosis in 1958 at the American Academy of Orthopaedic Surgeons Annual Meeting in Chicago. However, his presentation was met with skepticism from many of his peers, some of whom even questioned his continued membership in the professional society. Despite this initial resistance, Dr. Harrington remained steadfast in his belief that the technique had potential. He continued to refine his approach, working with materials such as stainless steel, and investigated novel surgical techniques and designs [5].

**Figure 5:**
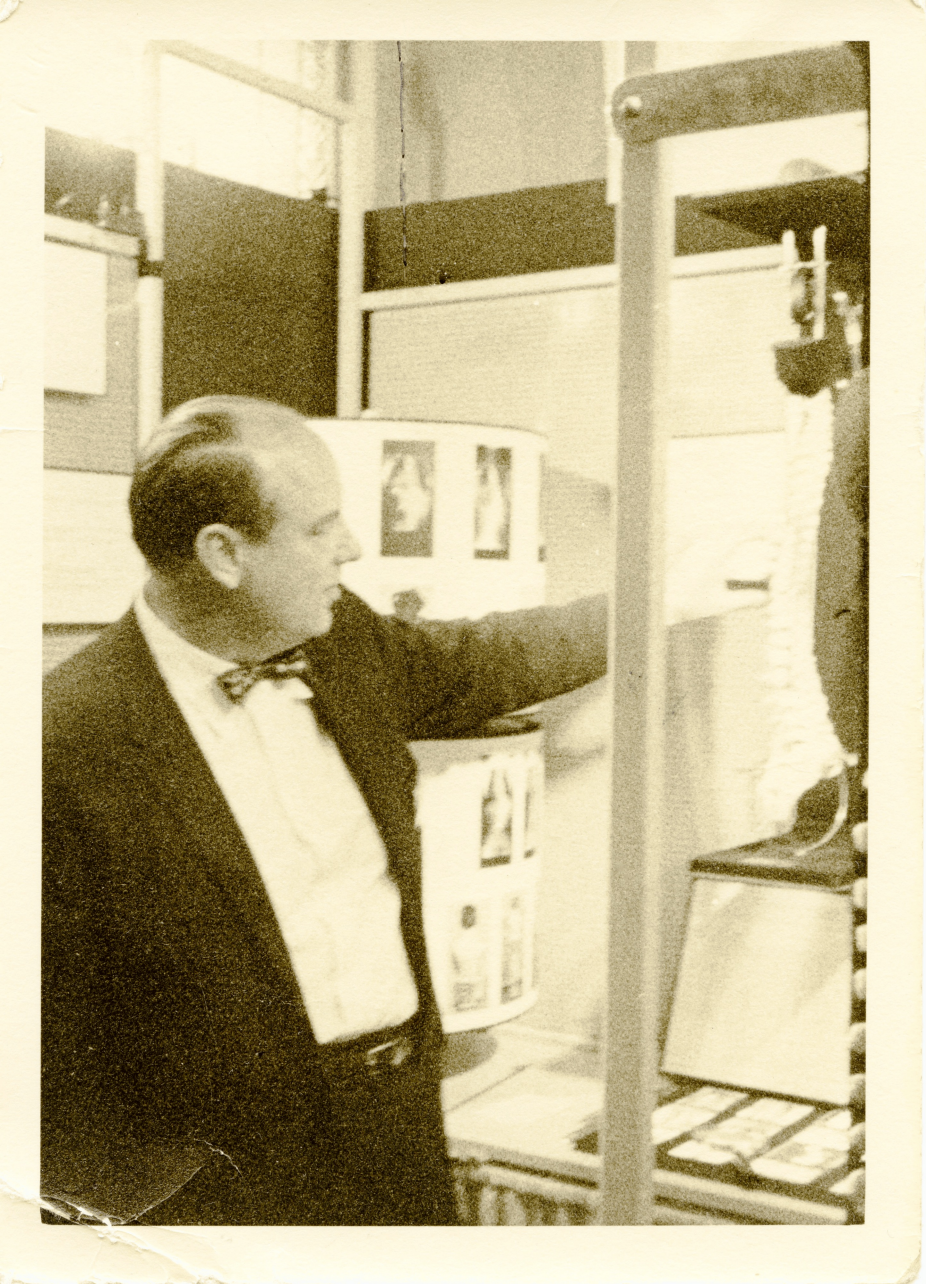
Dr. Paul Harrington explaining his system at an exhibition, circa 1959 Credit: Paul R. Harrington Archives, University of Kansas Medical Centre, Kansas City, KS.

**Figure 6:**
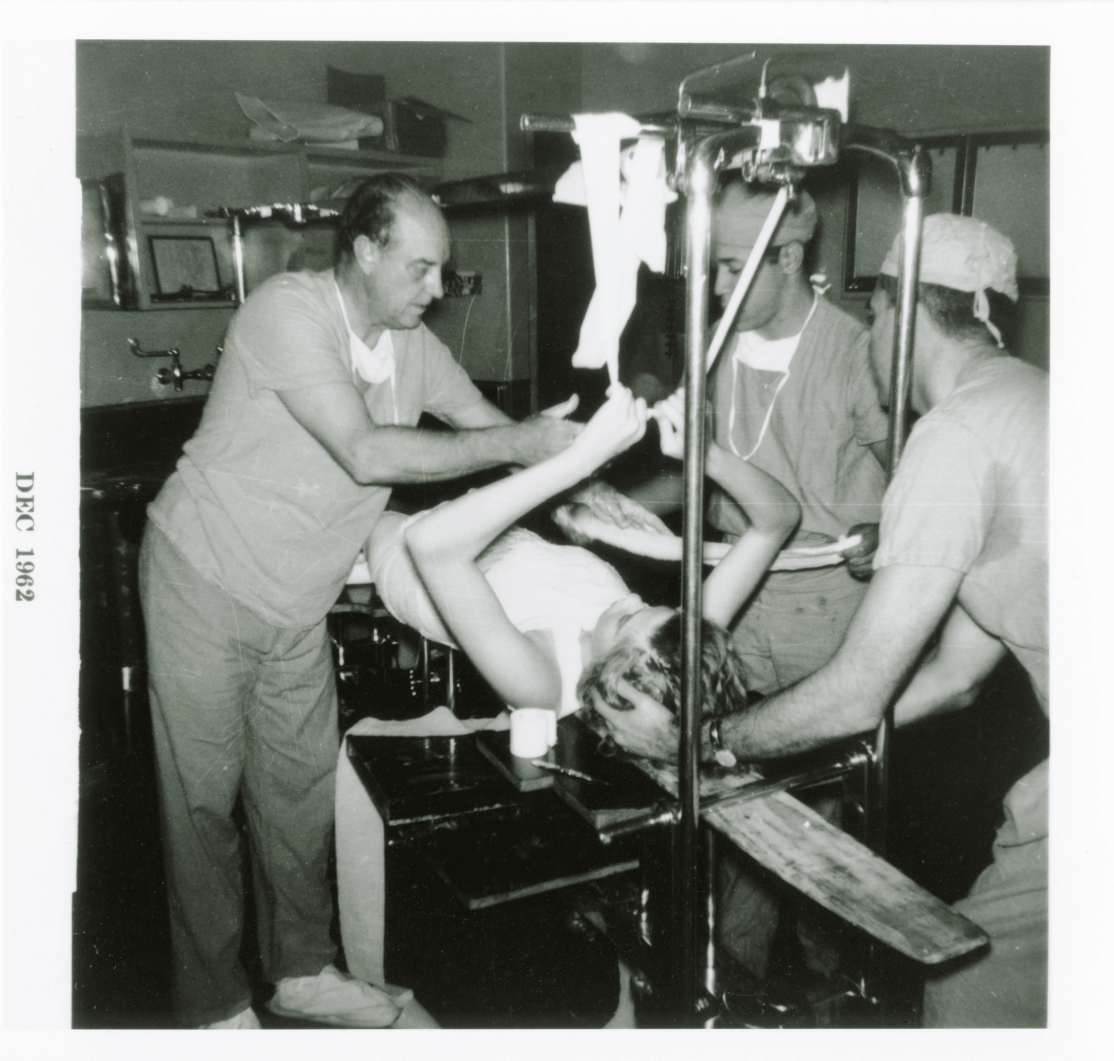
Dr. Harrington casting a patient, circa 1962 Credit: Paul R. Harrington Archives, University of Kansas Medical Centre, Kansas City, KS.

Eventually, Dr. Harrington's perseverance paid off. A commercial testing organization in Chicago and the Engineering Department at Rice University thoroughly tested his spinal instrumentation device. He also began collaborating with Zimmer Inc., a leading orthopaedic equipment manufacturer, to produce and distribute the Harrington rod system. This collaboration was crucial in bringing his innovation to a broader audience (Figure 5). Although the exact nature of their partnership is not fully detailed, it is believed that Dr. Harrington's use of Zimmer's leg braces for polio patients may have been the initial connection between them (Figure 6) [5].

The Harrington Instrumentation system, released in 1960, represented a significant breakthrough in spinal surgery. Zimmer Manufacturing, now Zimmer Biomet, successfully produced the instrumentation, and surgeons could obtain it after completing a training course. At the time of Dr. Harrington's death in 1980, it was estimated that over one million patients had benefited from the system [6].

To ensure the safety and effectiveness of the system, Dr. Harrington initially restricted the sale of the instruments to surgeons who had trained directly under him. This approach allowed him to maintain high standards of care and ensured that the surgeons using his system were well-versed in the technique. His commitment to improving patient outcomes and the innovative approach were later recognized when his work was featured in the Time magazine in November 1962 [7].

Until 1970, Dr. Harrington donated the royalties from the Harrington rod system to his research. Afterward, as his health began to decline due to peripheral vascular disease, the royalties provided him financial support. Dr. Harrington's relationship with Zimmer exemplifies the productive potential of the collaboration between industry and healthcare professionals, a model which has significantly advanced medical technology. Although contemporary guidelines might restrict such collaborations, the partnership between Dr. Harrington and Zimmer demonstrates how such relationships can lead to groundbreaking innovations in healthcare [8].

Legacy and influence

Dr. Harrington's innovations in spinal surgery laid the groundwork for subsequent advancements in spinal instrumentation. The Cotrel-Dubousset system was introduced in 1978, after the Luque rods along with sublaminar wires in 1977. They represented further progress in spinal deformity correction. These systems allowed for segmental correction and provided more rigid constructs, reducing the need for postoperative external immobilization. The development of pedicle screw fixation, which became widely accepted in the late 20th century, further improved the effectiveness of spinal surgery in correcting deformities and enhancing patient outcomes [9].

Dr. Harrington's novel approach to treating scoliosis focused on addressing the impact of asymmetrical spinal loads, a significant departure from the conventional methods of the time. He hypothesized that uneven facet loads led to asymmetrical growth, contributing to the progression of scoliosis. He designed internal supports to counter this, correct these imbalances, and promote normal spinal development. His early work involved manually crafting implant anchors, and with grants from the National Foundation for Infantile Paralysis, he advanced his research and developed prototypes with the help of orthotist Thorkild Engen.

He served as a Professor in both the Division of Orthopedic Surgery and the Department of Rehabilitation at Baylor College of Medicine. In 1973, he was honored with the Cora and Webb Mading Medal from the Texas Institute for Rehabilitation and Research (TIRR) Memorial Hermann (formerly the Institute for Rehabilitation and Research) and Baylor College of Medicine. That same year, he also received the prestigious Nicolas Andry Award from the Association of Bone and Joint Surgeons. Two years later, in 1975, the University of Kansas Medical Alumni Association bestowed him with the Distinguished Medical Alumni Award. From 1972 until his passing in 1980, Dr. Harrington collaborated with Marc Addason Asher to establish the Mary Alice and Paul R. Harrington Distinguished Professorship of Molecular Orthopedics at the Kansas University Medical College [10].

**Figure 7:**
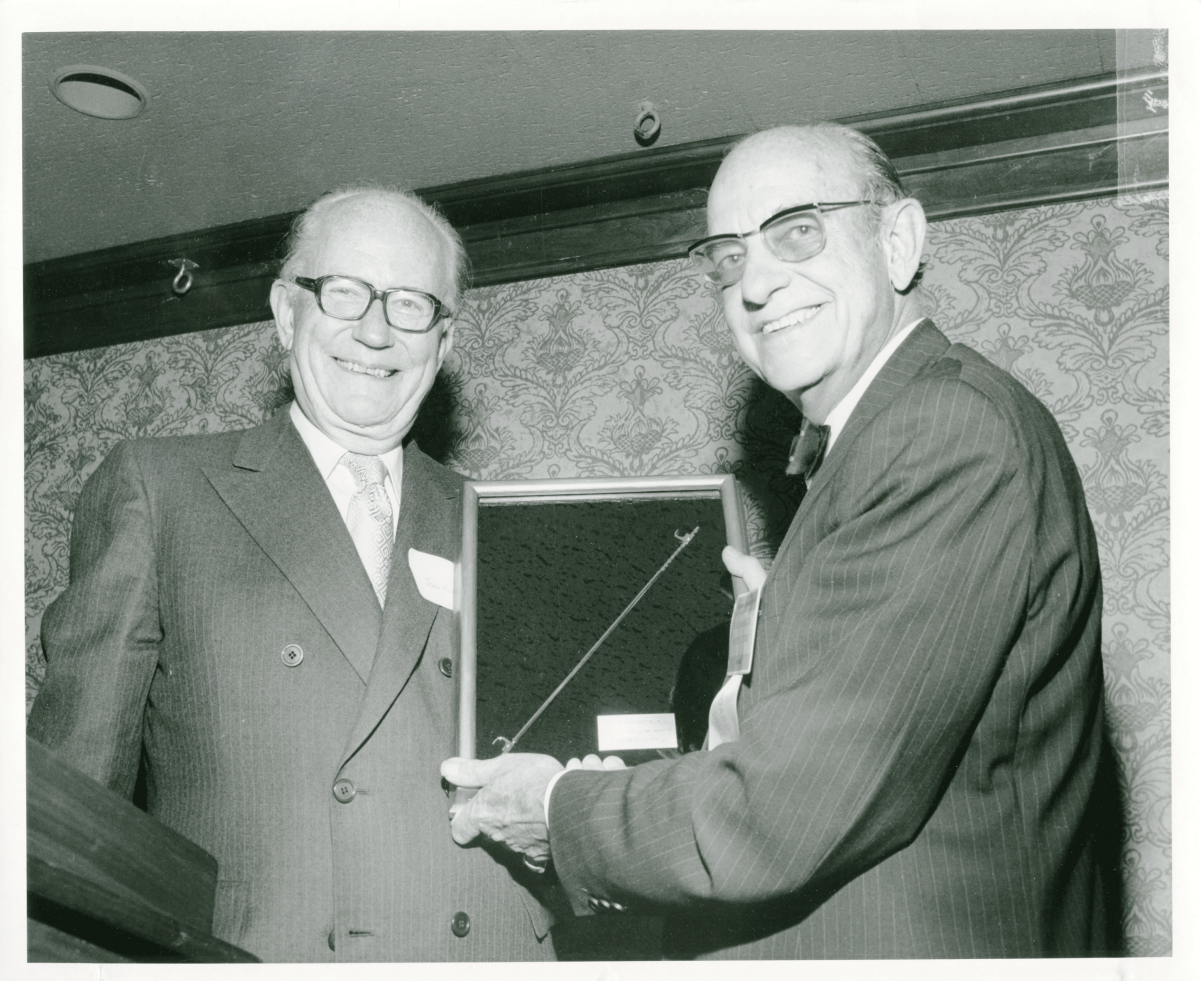
John Moe and Paul Harrington at the 9th Scoliosis Research Society annual meeting Credit: Paul R. Harrington Archives, University of Kansas Medical Centre, Kansas City, KS.

Dr. Harrington's contributions to spinal surgery extended beyond his instrumentation system. He played a central role in forming the Scoliosis Research Society, an organization dedicated to advancing the treatment of spinal deformities. He served as its president from 1972 to 1973, and his leadership helped establish the society as a leading authority in the field (Figure 7). Dr. Harrington also authored over 20 publications on spinal disorders, sharing his knowledge and experience with the broader medical community [11].

## Conclusions

Despite the advances in spinal instrumentation that followed, Dr. Harrington's work remains the cornerstone of modern spinal surgery. His approach to correcting spinal deformities, particularly in patients with neuromuscular conditions, continues to influence contemporary practice. While newer systems have surpassed the Harrington rod with respect to flexibility and effectiveness, the principles he developed are still relevant in treating complex spinal conditions.

Dr. Harrington's legacy is preserved through ongoing research, the continued use of principles he pioneered, and the recognition of his contributions by the medical community. His work during the polio epidemics, mainly focusing on the impact of spinal deformities on respiratory function, was groundbreaking and remains a critical area of study in spinal surgery. His dedication to improving patient outcomes, innovative spirit, and perseverance in the face of challenges have left an indelible mark on the field of orthopaedic surgery.
